# Potential Mechanisms of Transmission of Tick-Borne Viruses at the Virus-Tick Interface

**DOI:** 10.3389/fmicb.2022.846884

**Published:** 2022-05-05

**Authors:** Mahvish Maqbool, Muhammad Sohail Sajid, Muhammad Saqib, Faisal Rasheed Anjum, Muhammad Haleem Tayyab, Hafiz Muhammad Rizwan, Muhammad Imran Rashid, Imaad Rashid, Asif Iqbal, Rao Muhammad Siddique, Asim Shamim, Muhammad Adeel Hassan, Farhan Ahmad Atif, Abdul Razzaq, Muhammad Zeeshan, Kashif Hussain, Rana Hamid Ali Nisar, Akasha Tanveer, Sahar Younas, Kashif Kamran, Sajjad ur Rahman

**Affiliations:** ^1^Department of Parasitology, University of Agriculture, Faisalabad, Pakistan; ^2^Department of Epidemiology and Public Health, University of Agriculture, Faisalabad, Pakistan; ^3^Department of Clinical Medicine and Surgery, University of Agriculture, Faisalabad, Pakistan; ^4^Institute of Microbiology, University of Agriculture, Faisalabad, Pakistan; ^5^Section of Parasitology, Department of Pathobiology, KBCMA College of Veterinary and Animal Sciences Narowal, Lahore, Pakistan; ^6^Department of Parasitology, University of Veterinary and Animal Sciences, Lahore, Pakistan; ^7^Section of Parasitology, Department of Pathobiology, Riphah College of Veterinary Sciences, Riphah International University, Lahore, Pakistan; ^8^Department of Pathobiology, University of the Poonch Rawalakot, Rawalakot, Pakistan; ^9^Department of Parasitology, Cholistan University of Veterinary and Animal Sciences, Bahawalpur, Pakistan; ^10^Medicine Section, Department of Clinical Sciences, Collège of Veterinary and Animal Sciences, Jhang, Pakistan; ^11^University of Veterinary and Animal Sciences, Lahore, Pakistan; ^12^Agricultural Linkages Program, Pakistan Agriculture Research Council, Islamabad, Pakistan; ^13^Department of Zoology, University of Balochistan, Quetta, Pakistan

**Keywords:** ticks, immunity, tick-virus interaction, tick microbes, salivary glands

## Abstract

Ticks (Acari; Ixodidae) are the second most important vector for transmission of pathogens to humans, livestock, and wildlife. Ticks as vectors for viruses have been reported many times over the last 100 years. Tick-borne viruses (TBVs) belong to two orders (*Bunyavirales and Mononegavirales*) containing nine families (*Bunyaviridae, Rhabdoviridae, Asfarviridae*, O*rthomyxovirida, Reoviridae, Flaviviridae, Phenuviridae, Nyamiviridae*, and *Nairoviridae*). Among these TBVs, some are very pathogenic, causing huge mortality, and hence, deserve to be covered under the umbrella of one health. About 38 viral species are being transmitted by <10% of the tick species of the families *Ixodidae* and *Argasidae*. All TBVs are RNA viruses except for the African swine fever virus from the family *Asfarviridae*. Tick-borne viral diseases have also been classified as an emerging threat to public health and animals, especially in resource-poor communities of the developing world. Tick-host interaction plays an important role in the successful transmission of pathogens. The ticks' salivary glands are the main cellular machinery involved in the uptake, settlement, and multiplication of viruses, which are required for successful transmission into the final host. Furthermore, tick saliva also participates as an augmenting tool during the physiological process of transmission. Tick saliva is an important key element in the successful transmission of pathogens and contains different antimicrobial proteins, e.g., defensin, serine, proteases, and cement protein, which are key players in tick-virus interaction. While tick-virus interaction is a crucial factor in the propagation of tick-borne viral diseases, other factors (physiological, immunological, and gut flora) are also involved. Some immunological factors, e.g., toll-like receptors, scavenger receptors, Janus-kinase (JAK-STAT) pathway, and immunodeficiency (IMD) pathway are involved in tick-virus interaction by helping in virus assembly and acting to increase transmission. Ticks also harbor some endogenous viruses as internal microbial faunas, which also play a significant role in tick-virus interaction. Studies focusing on tick saliva and its role in pathogen transmission, tick feeding, and control of ticks using functional genomics all point toward solutions to this emerging threat. Information regarding tick-virus interaction is somewhat lacking; however, this information is necessary for a complete understanding of transmission TBVs and their persistence in nature. This review encompasses insight into the ecology and vectorial capacity of tick vectors, as well as our current understanding of the predisposing, enabling, precipitating, and reinforcing factors that influence TBV epidemics. The review explores the cellular, biochemical, and immunological tools which ensure and augment successful evading of the ticks' defense systems and transmission of the viruses to the final hosts at the virus-vector interface. The role of functional genomics, proteomics, and metabolomics in profiling tick-virus interaction is also discussed. This review is an initial attempt to comprehensively elaborate on the epidemiological determinants of TBVs with a focus on intra-vector physiological processes involved in the successful execution of the docking, uptake, settlement, replication, and transmission processes of arboviruses. This adds valuable data to the existing bank of knowledge for global stakeholders, policymakers, and the scientific community working to devise appropriate strategies to control ticks and TBVs.

## Introduction

Pests are causing damage to our lives. Several organisms act as vectors and transmit or cause diseases in the agriculture sector, humans, wildlife, and livestock. Ticks (Acari: Ixodida) are blood-sucking ectoparasites and act as vectors of pathogens infecting livestock, wildlife, and humans across the world (Bente et al., 2013; Pfäffle et al., 2013; Guglielmone et al., 2014; Monfared et al., 2015; de la Fuente et al., 2016; Sajid et al., 2017; Ghafar et al., 2020). Ticks and tick-borne diseases (hereafter abbreviated as TBDs) are known to decrease production below the genetic potential of livestock (Sajid et al., 2007). Ticks are ectoparasites of a wide range of mammals, reptiles, and birds (Karim et al., 2017). Even if not infected with tick-borne diseases (TBDs), ticks are responsible for direct damage to the skin and hides, cause allergy, irritation, and toxicosis, and can lead to decreased livestock productivity (Sajid et al., 2007). Over 80% of the world's cattle population is affected by tick-transmitted pathogens that cause diseases called TBDs (De Meneghi et al., 2016; Rodriguez-Vivas et al., 2018). They are one of the major management issues in Africa and the Americas, and are particularly important in Asia because of hot and humid climatic conditions (Moming et al., 2018; Rosà et al., 2018). The typical life cycle involving questing and infesting stages, host diversity, and inappropriate micro and macro-management lead to the successful settlement of these arthropod vectors (Soulsby, 1982). In some countries (e.g., Turkey), ticks have adapted themselves to wild animals, such as spur-thighed tortoises, which can act as reservoirs of infestation for contiguous livestock populations (Uslu et al., 2019).

It is a proven fact that ticks act as biological vectors of a wide range of causative agents of protozoal (e.g., babesiosis and theileriosis), bacterial (ehrlichiosis, borreliosis, Rocky Mountain spotted fever, and Q fever), viral (e.g., Crimean Congo hemorrhagic fever, and Powassan), and rickettsial (e.g., anaplasmosis) diseases and Lyme disease (Dantas-Torres et al., 2012; Mccoy et al., 2013; Pfäffle et al., 2013; Gharbi and Aziz Darghouth, 2014; Ghosh and Nagar, 2014; Guglielmone et al., 2014; Pantchev et al., 2015; Solano-Gallego et al., 2016; Rashid et al., 2019; Siddique et al., 2020). Ticks are known to infest a wide range of hosts, including humans, livestock, pets, and wildlife, and are considered the second most widely used vector for disease transmission among arthropods on the planet (again behind mosquitoes) (Monfared et al., 2015). Almost 898 tick species are recognized, belonging to three different families: Argasidae (soft ticks, 194 species), Nuttalliellidae (intermediate, 1 species), and Ixodidae (hard ticks, 703 species) (Latif et al., 2012). Among these, the Ixodidae is the most diverse, abundant, and dominant tick family from a One Health significance (Tsatsaris et al., 2016). The prevalence of tick-borne diseases (TBDs) has increased recently because of several biotic and abiotic factors (Estrada-Peña and de la Fuente, 2014, 2018; Estrada-Peña et al., 2017; Martina et al., 2017). Thus, ticks are among the major contributing factors to lowered production and mortality, and are the basic reason for economic losses in livestock around the globe (Grisi et al., 2014).

## Tick-Borne Viruses

Tick-borne viruses (TBVs), also known as tibo viruses, constitute various viruses that are transmitted successfully between two different environments. These are the host environment, the stable one, while the other is the opposite of stable, i.e., tick internal environment (Hubálek and Rudolf, 2012). Over time, the relationship between ticks and viruses has evolved. Consequently, the tick feeding cycle is synced with the viral life cycle, sculpting the evolution of tibo viruses (Sidorenko et al., 2021; Migné et al., 2022). Recent studies have illuminated the fact that tick cells undergo transcriptional changes while harboring viruses (Mansfield et al., 2017).

The history of TBVs links back to 1929, when the tick transmission of a flavivirus, the louping ill virus, accountable for encephalitis in sheep, was discovered (Bichaud et al., 2014; Shi et al., 2018). Later, in 1945, another TBV, the Crimean Congo virus, was confirmed in Soviet soldiers and local inhabitants of the Crimean Peninsula of the USSR (Zivcec et al., 2016). This led to a path for the subsequent discovery of heterogenous TBVs falling under two orders, *Bunyavirales* and *Mononegavirales*. These are divided into one DNA virus family (*Asfarviridae*) and eight RNA virus families (*Bunyaviridae, Rhabdoviridae, Orthomyxoviridae, Reoviridae, Flaviviridae, Phenuviridae, Nyamiviridae*, and *Nairoviridae*) (Nuttall, 2013).

**Figure 1:**
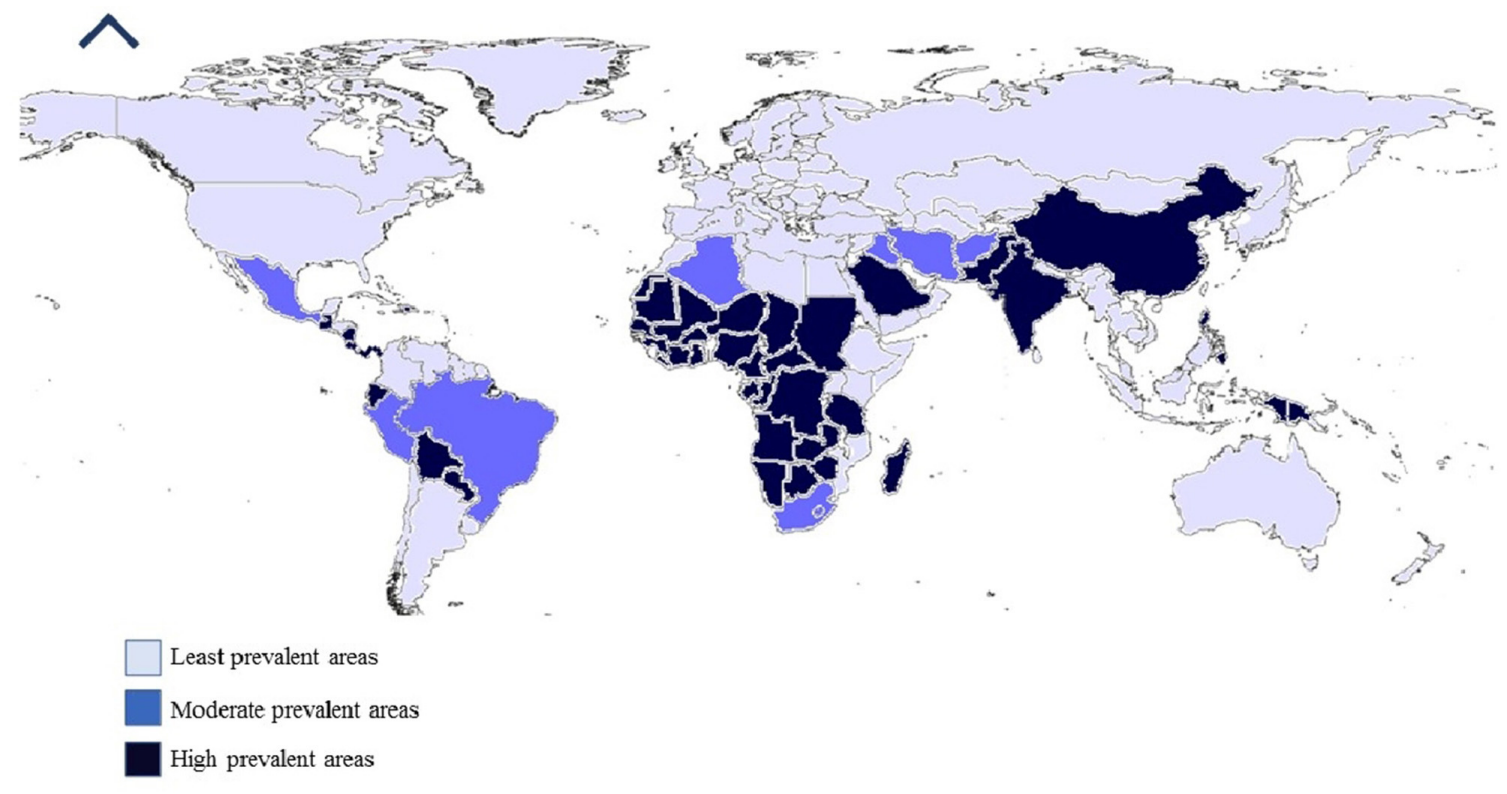
Worldwide prevalence of tick-borne viruses.

In the last decade, there was an emergence or re-emergence of tick-borne encephalitis virus that jeopardized public and animal health. There have been reports of TBVs in new geographical locations, a rise in several specific diseases, e.g., Possowan virus in America, and the occurrence of novel viruses, such as the Alkhurma virus, (a subtype of Kyasanur forest disease virus) (Burthe et al., 2020; Madani and Abuelzein, 2021; Yang et al., 2022), and deer tick virus (a subtype of POWV) (Hermance and Thangamani, 2018). These new viruses are placed in different families based on the latest molecular diagnostic techniques, resulting in major changes made in the families *Bunyaviridae* and *Rhabdoviridae* (Kazimírová et al., 2017) (Figure 1).

## Morphology Of Tick Salivary Glands

During tick-host interaction, host tissues (blood) and tick saliva pass through a common buccal canal. Tick saliva originates from branched and paired alveolar salivary glands, which are present anterolaterally and extend into the posterior sides of the body. From each salivary gland, a duct originates that enters a broad shallow tube, i.e., salivarium. This tube lies above the pharynx in the pharyngeal region valve and opens into the food canal at the anterior opening of the pharynx (Kemp and Tatchell, 1971).

## Anatomy Of Tick Salivary Glands Of Ixodidae

Complex alveoli are present in the Ixodid ticks' salivary glands, which are variable in numbers in female and male ticks, i.e., three in females and four in males (Balashov, 1972; Krolak et al., 1982; Barker et al., 1984).

### Type 1 Alveoli

The anterior region of the main salivary duct is attached with type 1 alveoli and may extend toward the posterior branches of the main duct. Type I alveoli contain granular cells.

### Type 2 Alveoli

Morphologically, types 2 and 3 alveoli are similar, and type 2 contains six types of granular cells, i.e., a,b, C-C4, which form reactions during staining procedures (Binnington, 1978). The morphology of an alveolus undergoes remarkable changes after several days of tick feeding but a change in number has not been reported. Enlargement of nuclei and the cytoplasm occurs, which, in turn, causes an increase in the overall mass of the alveolus. Along with this, abluminal interstitial cell enlargement also occurs, which forms a basal labyrinth during the feeding procedure. The granular materials present in the salivary glands of unfed ticks in the early feeding stage are absent during their final feeding stages. In the case of *Ixodes (I.) holocyclus*, type 2 female alveoli contain only two granular cell types (Šimo et al., 2013).

### Type 3 Alveoli

The most abundant type of alveoli in ticks' salivary glands are type 3 alveoli, which contain three granular cell types (d, e, and f), along with some granular cells. Type 3 alveoli are located in the posterior and peripheral regions of the glands (Binnington, 1978). The f cells of type 3 alveoli undergo cell transformation during the feeding process. The proliferation of cell plasma membrane occurs, and hypertrophy of abluminal interstitial cells and an increase in the number of mitochondria result in the formation of the basal labyrinth. Transformed f cells form a complex mass of plasma membranes and mitochondria, which is a common feature for fluid-transporting epithelia (Fawcett et al., 1986). An increase in the size of type 3 alveoli suggests that the transportation of the bulk of excreted fluid has taken place during the feeding process. In male ticks, the development of abluminal cells and f-cells occurs to a lesser degree than in female ticks (Coons and Lamoreaux, 1986). Only one common ad-luminal cell is present in types 2 and 3 cells that line the alveolus lumen in a web-like fashion (Labuda et al., 1993). In type 3 alveoli, the main function of ad-luminal cells is the same as that performed by myoepithelial cells, i.e., expansion of fluid-filled alveolus causing the ejection of fluid from the lumen to the salivary ducts and then outside the ducts (Kim et al., 2014).

### Type 4 Alveoli

Type 4 alveoli are only present in males along with abluminal and ad-luminal cells (Binnington, 1978). Only one granular cell type is present in type 4 alveoli, i.e.g., which is filled with secretion granules during the feeding process (Fawcett et al., 1986).

## Anatomy Of Tick Salivary Glands Of Argasidae

Argasids or soft ticks' salivary glands are less complex as compared to those of hard ticks and contain only two types of alveolar acini i.e., I and II (El Shoura, 1987). In soft ticks, coxal organs are involved in fluid secretion instead of salivary glands, and the change in salivary gland morphology during feeding is minor as compared to Ixodidae **(**Kaufman and Sauer, 1982).

### Type 1 Alveolar Acini

Type I alveolar acini are connected to the anterior region of the main salivary duct through short alveolar ducts. Cell types and ducts present in argasid type 1 alveoli are like Ixodid type 1 alveoli (El Shoura, 1987).

### Type 2 Alveolar Acini

In the case of type 2 alveoli, three granular cells are present, i.e., a, b, and c. The fourth type of cell, d, is also reported in *Ornithodoros* (*O.) savignyi* (Mans et al., 2004). The lumen of alveoli leads to a chitinous alveolar duct, which lacks a complex valvular structure compared to ixodid alveoli (Roshdy and Coons, 1975). Canaliculi formation occurs in argasid ticks but not to the extent observed in type III alveoli of Ixodid ticks (El Shoura, 1987).

## Development And Degeneration Of Salivary Glands

In newly hatched larvae, extremely small salivary glands are present, and only ducts are distinguishable. Few alveoli begin to develop in older Ixodid larvae, e.g., types 1, 2, and 3 alveoli are observed in larvae of *Haemaphysalis* (*Hae.) spinigera*. Type 4 alveoli are not distinguishable in larval stages (Ullah and Kaufman, 2014). The size of salivary glands enlarges during the larval feeding process, and when engorged larvae drop from the host, degeneration of salivary gland alveoli occurs. However, salivary ducts form branch ducts that terminate in small alveoli containing undifferentiated cells (Nodari et al., 2012). Salivary alveoli continue to increase in numbers and differentiate into types 1, 2, and 3 alveoli in nymphal stages, while fully differentiated types of salivary gland alveoli are present in molted adults (Esteves et al., 2017).

During tick feeding, a 25-fold increase in mass, protein content (stimulation of mRNA synthesis and new protein expression), and size of salivary glands takes place (Tirloni et al., 2017). Further development of salivary glands occurs after the mating process, and mRNA and protein synthesis rates become double during this phase. In mated rapid-feeding female ticks, higher ATPase, Na+, K+, and adenylate cyclaseactivities and fluid secretion rate are present (Yu et al., 2017). Different hemolymph-borne factors are involved in controlling these changes in salivary glands during feeding and mating phases, e.g., an applied juvenile hormone partially stimulates protein synthesis and ATPase, Na+- and K+- activity in *Amblyomma* (*A.) americanum* (Kim et al., 2016).

## Composition And Function Of Tick Saliva

Saliva is mostly water-derived during the blood meal process and, as in the Ixodid group, most of the blood meal is taken during the last 12–24 h of feeding, so the excess amount of saliva is produced during the final engorgement stage (Šimo et al., 2014). About 1-ml volume of saliva is secreted by large tick species (Koči et al., 2014). During blood feeding, extra water is secreted, so ions are balanced by secreting hypo-stomatic saliva containing 70% water and ions taken up during blood-feeding (Kim et al., 2017).

## Cement

The secure attachment of ticks to the host body is due to cement proteins, which result in a prolonged feeding period (Suppan et al., 2018). In the case of soft ticks (fast feeder), adults, and nymphal stags penetrate deep into the skin of the host and make some strong unnecessary attachment, while in hard ticks, all slow Ixodid feeders use cement and enhance protein production to ensure secure attachment by enlarging tick-host association. Types 2 and 3 alveolus cells d and e are involved in cement production (Mans and Neitz, 2004). Cement protein contains some lipid and glycol proteins (Leal et al., 2018). A recent proteomic analysis of a cement cone has provided information regarding the presence of metalloproteases and serine protease inhibitors in *A. americanum* (Bullard et al., 2016). Cement proteins also have antigenic properties and contain a 90-kDa polypeptide in d and e cells of type 3 alveolus cells, and some of them can be considered potent anti-tick vaccine candidates (Suppan et al., 2018). When a tick attaches to the surface of the host, the cement protein forms a cement cone around tick mouthparts and allows for firm attachment to the host's skin and protection against the host's immune system. A cement cone is also involved in playing an antibacterial role (Suppan et al., 2018). Cement cone production is a specialty of the Ixodid group and differs in composition, size, and shape among the members of this group (Leal et al., 2018). Glycine-rich proteins, such as those found in cement cones, are normally biologically inert and non-immunogenic; however, the use of cement proteins as a vaccine protects mice from lethal tick-borne encephalitis virus. These glycine-rich proteins also play a role in tick embryo development (Suppan et al., 2018).

## Enzymes And Enzyme Inhibitors Present In Tick Saliva

Tick saliva is a colorless, hypertonic, alkaline substance. Histochemical analysis of tick saliva has reported the presence of triacylglycerol, aminopeptidase, carboxylic ester hydrolases, and lipases (Bullard et al., 2016). Transcriptomic studies have revealed more than 500 different proteins and peptides further divided into different multigene groups, i.e., metalloproteases, lipocalins, Kunitz-domain proteins, some unique proteins found only in ticks, and some ancestral protein families that are present in Ixodid, argasid, and *Nutailella* (Mans et al., 2016). Esterases are also found in larvae of *B.microplus* causing a hypersensitivity reaction in cattle that are previously exposed to ticks; they hydrolyze cholesterol esters in the mast cell membrane, resulting in increased vascular permeability and release of pharmacologically active compounds, i.e., hyaluronidase. The kininase present in tick saliva deactivates the action of bradykinin (a pain-causing mediator) at the feeding site and enhances the tick feeding process (Mulenga et al., 2013).

Members of the Ixodid group contain proteins that inhibit the production of proteolytic enzymes, i.e., plasmin, trypsin, porcine kallikrein, and chymotrypsin (Štibrániová et al., 2013). The metalloprotease proteins of ticks have been identified in saliva, ovary, and midgut, and play an important role in tick blood uptake, vitellogenesis, blood digestion, and innate immunity (Kotál et al., 2015a). Metalloproteases have been identified in diverse tick species, e.g., *A. maculatum, Rhipicephalus (R.) samguneus, I. scapularis, R. microplus, I. ricinus*, and *A. americanum* (Ali et al., 2015a,b; Chmelar et al., 2016a). Ticks contain several protease inhibitors that play a role in tick-host interactions (Chmelar et al., 2016b). Four major groups of protease inhibitors are present in ticks: trypsin inhibitors, serpins, and Kazal domain and Kunitz domain cysteine protease inhibitors (Parizi et al., 2018).

Serine protease inhibitors are involved in the production of antimicrobial peptides, digestion of blood, and innate immunity (Meekins et al., 2017). Serine protease inhibitors also bind with different protease and non-protease ligands, including maspin, nexin-1, kallistatin, and anti-chymotrypsin. Different tick species were screened for serine protease inhibitors and reported in *I. ricinus, D. variabilis, R. microplus, I. scapularis, A. variegatum, Hae. logicorns, A. americanum, R. appendiculatus*, and *A. hebraeum* (Kim et al., 2014). Characterization of some serine protease inhibitors has been conducted, and they were found to play a role in host defense mechanisms through pro-inflammatory cytokine production (Wikel, 2013; Valdés, 2014).

Another large group of tick protease inhibitors is that of cystatins, which modulate vertebrate biological processes, i.e., immunity, antigen presentation, phagocytosis, apoptosis, and hemoglobin digestion and regulation (Chmelar et al., 2016b). The cystatin group is further divided into four subgroups, i.e., types 1, 2, 3, and 4 cystatins (Kazimírová and Štibrániová, 2013). In ticks, types 1 and 2 cystatins are present, and type 1 was first isolated from *R. microplus* causing inhibition of vitelline by degrading the cystatin endopeptidase, and plays an immunomodulatory role (Schwarz et al., 2012). Different cystatins are reported on various tick species, i.e., *Hae. longicornis, A. americanum, I. ovatus, I. scapularis*, and *R. microplus*, and on a soft tick, *Orintodoros moubata* (Chmelar et al., 2017). Type 2 cystatins were characterized in *I. scapularis* and play an important role in the transmission of tick-borne pathogens by interfering with interferon-mediated immune responses and increasing the multiplication of tick-borne viruses in bone marrow dendritic cells (Chen et al., 2014). Various other cystatin forms are characterized by tick species, and interference with their actions results in the impaired feeding process, higher mortality, immunosuppressive effects, and block attachment, and are promising anti-tick vaccine candidates (Kotsyfakis et al., 2008; Chmelar et al., 2017).

## Prostaglandins

Prostaglandins have been identified in tick saliva and possess anti-inflammatory, hyperemic, and immunosuppressive activities (Chmelar et al., 2012). Different forms of prostaglandins are identified in ticks' saliva, i.e., F_2α_, A_2_/B_2_, prostacyclin (I_2_), and D_2_; among these, A_2_/B_2_ is considered to be derived from PGE2 because of the alkaline nature of tick saliva (Carvalho-Costa et al., 2015). Arachidonic acid and its derivatives act as precursors for salivary gland prostaglandins, e.g., endocannabinoids in *A. americanum*. Arachidonic acid is 8% of total fatty acids in partially fed tick salivary glands compared to the 2% in unfed ticks and increases more than any other fatty acid (Gao et al., 2016). Most vertebrates are capable of synthesizing arachidonic acid from linoleate by desaturation and elongation reactions, but in the case of ticks, females have the capability to synthesize degenerative fatty acids, but desaturation ability is lacking in ticks, and the stearoyl CoA desaturase gene is present in ticks. Phospholipase PLA_2_ activity is related to arachidonic acid release. Dopamine was also found associated with an increase in free arachidonic acid by stimulating PLA_2_ by the opening of voltage-dependent Ca2+ channel (Kannangara and Patel, 2018).

## Exosomes And Known Unknowns

Tick saliva also contains some exosomes, i.e., microRNA (miRNA) in the Ixodid species, and is involved in change in exosomal origin (Díaz-Martín et al., 2015; Hackenberg et al., 2017; Rodriguez et al., 2018). A transcriptomics analysis revealed the presence of some known unknowns in tick saliva, e.g., in *D. andersoni*, 677 proteins are identified, and out of this, 80% are of unknown function (Mudenda et al., 2014). It is also probable that tick saliva also contains some unknown unknowns, which are likely to reveal further interesting information regarding the saliva of ticks.

## The Function Of Tick Saliva In Controlling Host Response

When a tick bites, it causes activation of coagulation factor XII and bradykinin release, which cause host pain sensation; however Ixodid ticks destroy the bradykinin bymetalloprotease enzyme angiotensin-converting enzyme (ACE) (Chmelar et al., 2012). Two types of ACE have been identified in *A. maculatum* (Jelinski, 2016). Ticks also use lipid mediators (endocannabinoids) as an analgesic to hide their presence, and, along with this, other analgesic mediators are found: adenosine and miRNA (Hackenberg et al., 2017).

Vasoconstriction is another phenomenon that occurs during tick bite, and Ixodid ticks use prostaglandins and adenosine as counter agents against vasoconstriction (Chmelar et al., 2012). Other proteins involved in vasoconstriction are apyrase, serotonin-binding salivary proteins, histamine-binding proteins, and phenylalanine-rich peptides, which may modulate vascular permeability (Pekáriková et al., 2015).

Platelet aggregation and activation are controlled during tick bite, by the tick releasing, *via* the saliva, apyrases, thrombin inhibitors, arginine-glycine-aspartate motif, and serotonin binders, which break down the platelet activation agonist ADP, damage the cells, and, ultimately, neutralize the platelet aggregation agonist (Tang et al., 2015; Yun et al., 2016). After platelet plug formation, the secondary phase of hemostasis occurs, i.e., coagulation factor assembly, which leads to fibrin plug formation (Palta et al., 2014). Tick saliva contains serine protease inhibitors as anti-coagulants which target the thrombin and coagulation factor FXa (Blisnick et al., 2017) along with these Kunitz domains also contain thrombin inhibitors e.g., *O. moubata* contain ornithodorin, variegin in *A. variegatum*, Salp 14 in *I. scapularis* saliva, and ixonexin (tail peptide) in *I. scapularis* (Thompson et al., 2017; Assumpção et al., 2018). This multi-targeted approach by ticks allows for successful control of the coagulation process by interacting with the host coagulation cascade.

Mast cells containing pro-inflammatory compounds are present in abundance in host skin and act as the first line of defense against ticks (Wernersson and Pejler, 2014). When ticks attach to host skin, they activate mast cells, which results in degranulation, and the release of their contents into the extracellular environment, starting bioactive compound *de novo* synthesis. Ticks use lipocalins, Kunitz-type proteins, to control histamine, serotonin, and tryptase activity, stabilize mast cells, and prevent *de novo* synthesis (Schuijt et al., 2013). Sialostatin L targets IRF-4-dependent transcription in mast cells, resulting in interleukin-9 suppression (Klein et al., 2015). Pro-inflammatory cytokines (i.e., IL-1, IL-6, IL-8, and TNF) are produced by the host, and ticks inhibit these cytokines by capturing the ligand using cytokine binders called evasions, and about 265 evasions have been identified in different tick genera (Hayward et al., 2018). In *A. variegatum*, the salivary peptide amphiregulin inhibits cytokine production (Tian et al., 2016). The complement system is the main trigger for inflammation, and about 40 proteins take part in this phenomenon. During tick bite, the complement system is inhibited by the different complement inhibitors present in tick saliva that is produced during feeding and stored in the granular acini (Jore et al., 2016; Perner et al., 2018).

Ticks can control host immune responses by producing immunomodulators that target the host acquired and innate immune system (Kotál et al., 2015a; Wikel, 2018a). In *R. appendiculatus*, 64 TRP proteins cross-react with epitopes in tick midgut (Kotál et al., 2015b; Chmelar et al., 2016b). Dendritic cells are known as immune sentinels and sense danger and send information to other immune cells and contribute to both adaptive and innate immunities (Heath and Carbone, 2013; Austyn, 2017). In metastriate Ixodid ticks, lipocalin proteins are available that target dendritic cells, e.g., japanin (Preston et al., 2013), while in prostriate ticks, the sialostatin L group, cystatin protease inhibitors, are available for dendritic cell control, e.g., Salp 15 that inhibits CD4^+^ T cell and dendritic cell activation (Carvalho-Costa et al., 2015; Kotál et al., 2015b; Tomás-Cortázar et al., 2017). Regulatory T cells are also controlled by Salp 15 by the production of immunosuppressants, e.g., adenosine (Tomás-Cortázar et al., 2017). Salp 15 also affects the ability of B cells to produce antigen-specific antibodies, and direct inhibitors of B cells are also found in tick saliva, e.g., B cell inhibitory proteins in *I. ricinus* and B cell inhibitory factors in *Hyalomma* (*H.) asiaticum* (Páleníková et al., 2015).

Dynamic changes in salivary bioactive compound activity are correlated with host responses, cellular and chemical mediators, gluttony, and sex (Heinze et al., 2014). In adult *D. andersoni* females, it was found that within 2–5 days of the start of the feeding process, a total of 372 proteins can be identified, and among these, almost 140 were identified on day 2 and 165 on day 5 (Mudenda et al., 2014). Expression of saliva genes was recorded to be higher in female ticks than in males, which reflects the goals of feeding females to attain maximum blood meal size and increase egg production (De Castro et al., 2017). Transcriptomics studies have confirmed that salivary gene expression is variable as feeding progresses in *I. ricinus* females (Perner et al., 2018). For changes in saliva compositions, the term “sialome switching” is used. This change may be attributed to feeding environment changes, i.e., change in host type and host immune system (Karim and Ribeiro, 2015). Studies on saliva time regulation are limited; possibly, epigenetic regulation, chromatin remodeling, and histone modification are involved (Kotsyfakis et al., 2015; Cabezas-Cruz et al., 2016). Salivary gland acini granules are also involved in tick blood-feeding dynamism. Immunoglobulin binding proteins specific for males are stored in unfed *R. appendiculatus* male type IV acini, cement protein in type 3 acini in *D. variabilis*, and migration inhibitory factor (MIF) in *A. americanum* (Perner et al., 2018). This indicates that tick saliva is ready to start its action as soon as ticks attach and start feeding, and the contents of early salivary gland granules help to elucidate tick-host interaction at the feeding site and are early-stage targets for anti-tick vaccine development.

## Transmission Dynamics Of Viruses In Ticks

**Figure 2:**
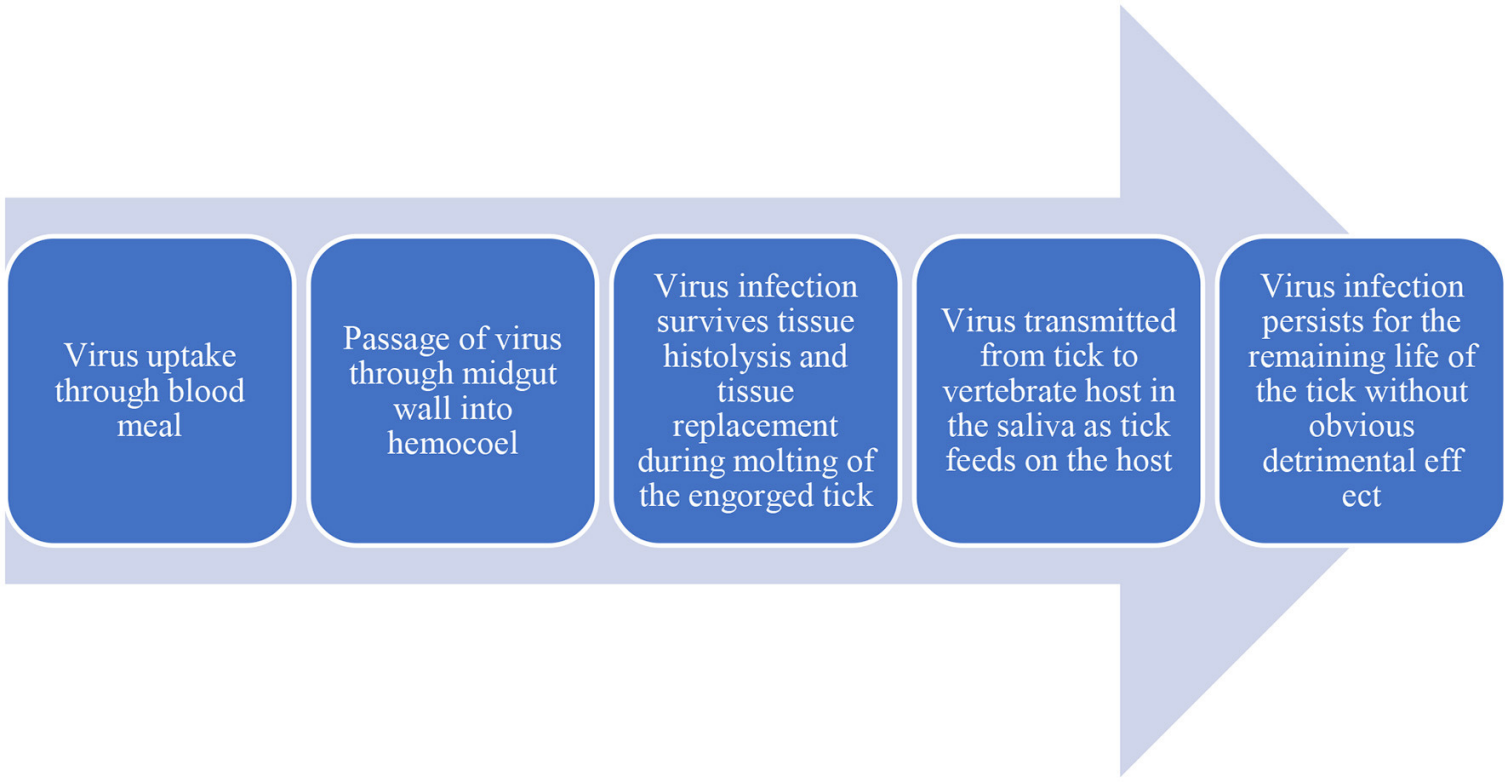
Flowdiagram depicting the virus flow in Host and Vector.

**Figure 3:**
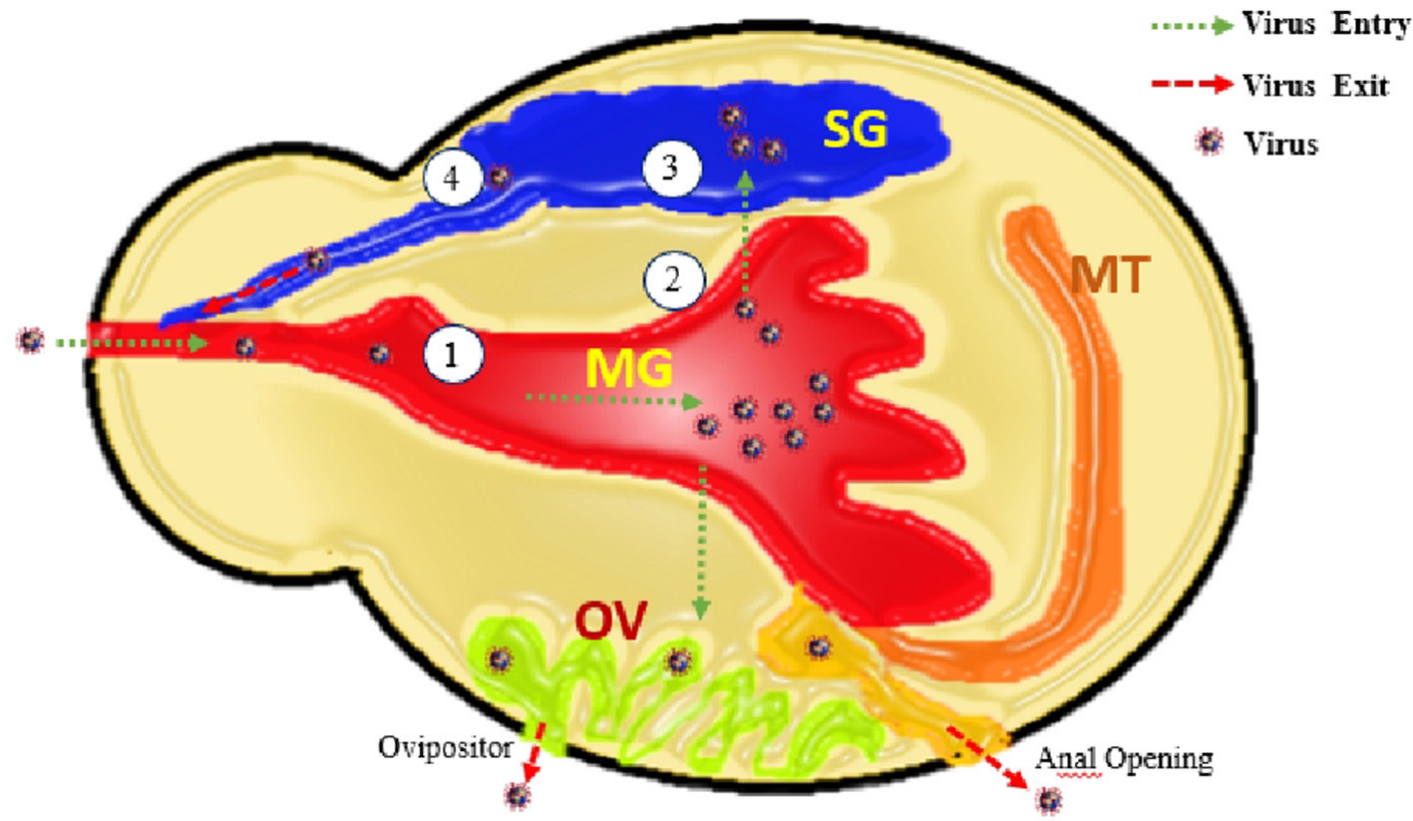
Elaborating the entry of virus through mouth opening along with bleed meal into mid gut (MG), virus multiplication and transfer into ovaries (OV), Salivary glands (SG), and anal opening. From these routes virus shed with saliva, transovarial (with next progeny of ticks), and anus. Along with these four barriers 1. Entry of virus into MG-cell, 2. Exit of virus from midgut cells, 3. Entry of virus into SG-cells (acini), and 4. Exit of virus from MG-cells.

Vector-borne viruses (VBVs) exhibit biological transmission: they enter into the vector, infect, and replicate before reaching the vertebrate host. Following the entry of a virus into the midgut of ticks, it must escape from the midgut and reach the tick salivary glands from which it will be transmitted to the vertebrate host (Šimo et al., 2017). This is described as an extrinsic incubation period as the virus remains inside the vector. Movements of the virus within a vector (midgut to salivary glands) are life-threatening for viruses because of various potential barriers: midgut infection, midgut escape, salivary gland infection, and salivary glands escape (Kazimírová et al., 2017) (Figures 2, 3).

More precisely, at the cellular level, the virus may remain unable to cross the cell membrane for entry into the cytoplasm, or the virus may replicate inside the cell following entry but is incompetent to come out of the infected cell (Dou et al., 2018). The intrinsic ability of a tick to become infected, support replication, and ultimately, transmit a tick-borne virus is genetically determined and influenced by environmental factors. Likewise, the ability of a tick-borne virus to infect, replicate, and be dispersed by a tick is both determined genetically and influenced by extrinsic factors. At one level, vector competence is determined through genotype-by-genotype interactions (Althouse and Hanley, 2015). In this sense, the outcome of infection depends on the interplay between the products of 2 genomes, the so-called virus–vector interactome. However, molecular interactions between tick-borne viruses and their tick vectors are yet to be explored. For example, we know little about the role of RNA interference (RNAi) in ticks and, in particular, whether it acts as an innate antivirus immune response modulating virus infection (Kurscheid et al., 2009; Kazimírová et al., 2017). Evidence in mosquitoes indicates that the RNAi pathway modulates arboviral infections, for example, by acting as a gatekeeper to the incoming viruses at the midgut, by minimizing the intensity of the viral infections, and reducing the spread of viruses from the midgut to secondary tissues (Khoo et al., 2010). It seems likely that a similar phenomenon occurs in ticks (Kazimírová et al., 2017).

## Midgut Infection Barrier

Evidence of a midgut infection barrier has been reported in experimental studies with *Rhipicephalus appendiculatus* and *Amblyomma variegatum*, two tick species that are adept vectors of the Thogoto virus but are not competent for the Dhori virus (Gondard et al., 2020). When larvae and nymphs were fed on virus-infected hamsters, the Thogotovirus settled and replicated within the ticks and was subsequently transmitted when the succeeding adults fed on uninfected hamsters. In contrast, both tick species were refractory to infection by the Dhori virus when they fed on hamsters infected with this virus, with infectivity in the engorged ticks disappearing in 2–6 days. However, when the Dhori virus was inoculated into the hemocoel of engorged nymphs, effectually bypassing the midgut, the virus survived transcardially and was transmitted during the feeding of infected ticks. Thus, the midgut of *R. appendiculatus* and *A. variegatum* appear to be a barrier to infection by the Dhori virus but not by the Thogoto virus. As the Thogoto virus and the Dhori virus are members of the same virus genus and have similar infection strategies, the most likely reason for the variation in vector-species specificity lies in the sequence diversity of viral surface glycoproteins (Gondard et al., 2020). If this is the case, specific surface receptors might be existing on the surface of tick midgut cells to which the Thogoto virus binds *via* its glycoprotein but are not recognized by the Dhori virus. Alternatively, the Thogoto virus might have evolved a mechanism for evading the defense mechanism of *R. appendiculatus* and *A. variegatum* that is efficient against the Dhori virus.

Studies on African swine fever virus have also demonstrated the importance of virus replication in the midgut for successful infection of its vector, *Ornithodoros porcinus* (Nuttall, 2019b). A Malawi strain of the virus failed to replicate successfully in midgut epithelial cells of ticks exposed orally to the virus, although the virus replicated successfully in other cell types. Moreover, a different virus strain was infected and replicated successfully under the same experimental conditions. The results suggest that missing or defective genes in the Malawi strain might account for the failure of the virus to replicate successfully in midgut epithelial cells, although why this should be the case for midgut cells and not appear for other cell types is a conundrum (Lledó et al., 2020). Compared with evidence of a midgut infection barrier based on experimental studies with the Dhori and Thogoto viruses, African swine fever virus data suggest that there might be different types of midgut infection barriers in ticks (Rock, 2021).

One type of midgut barrier might be provided by the unusual way in which ticks digest their blood meal. Unlike insects, in which blood meal digestion is extracellular, ticks are heterophagous: intracellular digestion of blood meal takes place in midgut cells. Several insect-borne viruses depend on proteolytic conditions in the insect gut lumen to cleave a surface protein and expose the virus receptor that initiates infection of the vector (Talactac et al., 2021). The absence of such proteolytic enzymes in tick lumen could provide a highly efficient barrier to infection by viruses that require cleavage of a surface virus protein to initiate infection. If the process of blood meal digestion in ticks is an efficacious barrier to virus infection, arboviruses that can infect ticks are likely to have evolved an outer surface structure that differs significantly from that of their genetic relatives that are not transmitted by ticks. There are some data to support this hypothesis: (i) striking size variations in outer surface proteins of midge-transmitted arboviruses relative to the tick-transmitted arbovirus Broadhaven virus (Nuttall, 2013) and (ii) similarly pronounced differences in surface glycoproteins of influenza viruses relative to their tick-borne relatives (Shi et al., 2018).

However, the three-dimensional structures of the flavivirus that envelope proteins of tick-borne and mosquito-borne flaviviruses appear similar, although this similarity might reflect the common fusion role of this protein after entry into cells (Lemasson et al., 2021). One factor in the infection process that is usually overlooked is the state of a virus within the blood meal of its vector, whether as an extracellular virion (virus particle) or as an infected cell that may potentially be imbibed in the infected blood meal of a feeding tick. If a cellular rather than an extracellular viral inoculum is more effective in establishing infection in the tick vector, this might in part explain the efficiency of non-viremic transmission. Besides the state of a virus in the blood meal (whether “free” or within host cells), the timing of virus uptake also might be a critical factor in determining whether a virus infects a tick. This is because, like hematophagous insects, ticks produce a peritrophic membrane or glycocalyx on the apical surface of the midgut epithelium some hours after the commencement of feeding (Bhowmick and Han, 2020). The chitin-enriched covering potentially presents a formidable barrier to the infection of midgut epithelial cells by viruses. Studies on mosquitoes have shown that virions ingested in the viremic blood meal acquired from chickens infected with western equine encephalitis virus concentrate adjacent to the midgut epithelium. In contrast, when ticks fed on an artificial blood meal containing the virus, the disseminated virus was observed throughout the midgut lumen (Talactac et al., 2021). It waits to be revealed whether such concentration of virions occurs in the tick midgut and/or whether ingestion of infected cells rather than “free” virions helps the virus overcome the barrier presented by the peritrophic membrane. This suggests that viruses are unable to survive if they do not exit the midgut.

## Midgut Escape Barrier

The evidence of a midgut escape barrier in ticks is based on comparative studies on infection of *R. appendiculatus* nymphs infected with the Dhori virus or the Dugbe virus. The Dhori virus can survive for <4 days in *R. appendiculatus* nymphs following vector feeding on an infected host. The Dugbe virus can survive for at least 21 days after vector meal ingestion but remains unable to survive during molting and has no transmission through adult ticks. Following virus inoculation directly into the hemocoel, just like the Dhori virus, the Dugbe virus replicates and is transmitted by *R. appendiculatus;* explaining that, there are no barriers for the Dugbe virus to infect the salivary glands of *R. appendiculatus* as for the Dhori virus (Kazimírová et al., 2017; Nuttall, 2019a). The variation in survival dynamics recommends that *R. appendiculatus* reveals a midgut infection barrier to the Dhori virus and a midgut escape barrier to the Dugbe virus. However, the nature of the midgut escape barrier is unknown.

## Dissemination Barrier

Once a tick-borne virus has escaped from the tick midgut, it presumably passes through the hemocoel, where tissues and organs are immersed in hemolymph, the transport medium for hormones, nutrients, and immune effecter molecules. To migrate to the salivary glands while hiding from the tick's immune system, viruses, such as tick-borne encephalitis virus, African swine fever virus, and Dugbe virus, infect tick hemocytes (Talactac et al., 2021). An alternative route of dissemination is *via* the nervous system. However, although the Thogoto virus was recognized in the neural cortex of the synganglion, it was not apparent in nerve fibers, suggesting that dissemination through tick vector occurs *via* the hemolymph rather than a neural route (Grabowski et al., 2018). A dissemination barrier might exist in mosquitoes in which a virus is restricted to abdominal fat body cells, which play a role in insect immune responses (Lee et al., 2019). Their presence in ticks has not been described.

## Salivary Gland Infection Barrier

After reaching the salivary glands, a virus faces barriers like those of the midgut: (i) cell infection and replication and (ii) virus release. Although there are some records of virus detection in saliva, little is known about this critical stage of tick-borne virus transmission (Nuttall, 2019a). Experimental studies with the Thogoto virus and *Amblyomma variegatum* indicate that infection of the salivary glands might not be a precondition for transmission (Gondard et al., 2020); extracellular virus inoculated into the hemocoel was detected shortly afterward in the saliva of ticks. The result was consistent with previous observations that the transmission of Thogoto virus occurred within 24 h of tick attachment to a host, even though virus infection of the salivary glands was not detected until 7 days after feeding commenced. The mechanism of virus transfer from the hemocoel to saliva is unknown. Some proteins found in the hemocoel (e.g., host immunoglobulins) appear to be excreted in tick saliva even though tick salivary glands exclude smaller molecules, such as polyethylene and inulin (Brzezinski, 2019). If tick-borne viruses can pass from the hemocoel into saliva without requiring infection of the salivary glands, salivary gland infection and escape barriers as described for the mosquito-borne transmission of insect-borne arboviruses might not exist in ticks. More importantly, there might be processes by which tick-borne viruses can be transmitted rapidly to the vertebrate host, presenting a greater epidemiological risk to humans.

Interestingly, Thogoto virus-infected ticks secreted less saliva than uninfected ticks or ticks inoculated with the virus into the hemocoel (Nuttall, 2019a). Possibly, virus infection had a deleterious impact on the fluid secretory process. Alternatively, the virus might have stimulated a more vigorous secretion in infected ticks, which would result in lower saliva volumes collected during experimentation. The latter hypothesis is consistent with observations that the tick-borne encephalitis virus stimulates the aggressiveness of its tick vector, *Ixodes persulcatus* (Morozova et al., 2020).

## Salivary Gland Escape Barrier

Once a tick-borne virus has passed into the infected tick's hemocoel and survived molting, there might be a mechanism where the virus can pass into the saliva without having to overcome barriers to infection of the salivary glands. However, evidence of infection of tick salivary glands has been reported for several tick-borne viruses. For example, Dugbe virus infection was detected in discrete cells of type 3 salivary gland acini (Kramer and Tavakoli, 2021). A virus infecting the salivary glands has to be released into the salivary ducts to be transmitted in saliva. Little is known of the mechanisms of release of salivary proteins into saliva, let alone viruses. Similarly, the impact of the physicochemical properties of saliva on tick-borne viruses is unknown. There is no evidence that salivary proteins interact directly with virions, as reported for the tick-borne bacterium *Borrelia burgdorferi*. However, if tick saliva has a pH value in the range of 9–9.5, as some studies have indicated, the alkalinity of saliva could have a profound effect on the conformation of virions in tick saliva. For example, the icosahedral outer surface of the tick-borne encephalitis virus is steady in a limited pH range and opens when exposed to either acidic or alkaline circumstances (Šimo et al., 2017).

## Trans-Stadial Survival

After engorgement, immature ticks undergo ecdysis, and histolytic enzymes and tissue replacement create a potentially hostile environment for viruses. For example, the salivary glands undergo reabsorption and restoration during molting. Thus, an essential feature of a tick-borne virus is its ability to survive the molting period for the virus to be transmitted from its tick vector to a vertebrate host. Virus replication dynamics in ticks might indicate these changing environmental conditions (i.e., a fall in infectious virus titter followed by an increase in titter as a virus infects and replicates in replacement tissues) (Nazar et al., 2013; Yoshii, 2019; Migné et al., 2022). However, the replication of some viruses (e.g., Langat in *I. ricinus* and Thogoto virus in *R. appendiculatus*) does not follow these dynamics (Godsey et al., 2021; Hart and Thangamani, 2021). The conflicting results may be explained by various cell and tissue tropisms of tick-borne viruses in their tick vectors. For example, the Thogoto virus establishes infection in the synganglion, where presumably it is safe from the processes of tissue replacement (Nuttall, 2013, 2019b; Morozova et al., 2020).

Because the salivary glands experience reabsorption and rejuvenation during molting, salivary gland infection is expected to be a relatively late event in virus dissemination in tick vectors following the uptake of an infective blood meal. The actual timing of infection of the salivary glands appears to vary. Tick-borne encephalitis virus and Powassan virus infect tick salivary glands before the commencement of feeding; seemingly, they can be transmitted to the vertebrate of the host as soon as fluid secretion occurs (Morozova et al., 2020). In contrast, the Thogoto virus and the Dugbe virus amass in the salivary glands following the commencement of feeding (Nuttall, 2013), although in ticks infected in the earlier stage, the Thogoto virus is found in the salivary glands before blood-feeding (Nuttall, 2019a).

## Horizontal Transmission By Ticks

The principal route of transmission for tick-borne viruses is horizontal, from an infected tick to an uninfected definitive host and from an infected host to an uninfected tick. Classically, horizontal transmission from vertebrate to tick was suggested to depend on the level of viremia (virus circulating in the blood). It is now recognized that tick-borne viruses can be transmitted effectively even when an infectious virus is not detectable in the blood (Turell, 2020).

## Viremic Transmission

Arboviruses have been defined as “viruses that are maintained in nature principally, or to an important extent, through biological transmission between susceptible vertebrate hosts by hematophagous arthropods or through transovarial and possibly venereal transmission in arthropods: the viruses multiply and produce viremia in the vertebrates, multiply in the tissues of arthropods, and are passed on to new vertebrates by the bites of arthropods after a period of extrinsic incubation.” A crucial point in the WHO definition of an arbovirus is the production of viremia. In animals (including humans) infected by the bite of a tick infected with tick-borne encephalitis virus, the virus replicates first in the skin site of tick feeding and in lymph nodes that drain the site. Neutrophils, monocytes/macrophages, and Langerhans cells attracted to the tick feeding site become infected (Hermance and Thangamani, 2018). Viremia develops when a virus is carried *via* the lymphatics to the thoracic duct and into the bloodstream. Primary viremia seeds are extraneural tissues that support further virus replication and shedding of the virus into circulation. In studies on mosquito-borne viruses, the threshold level of viremia was defined as the lowest amount of virus capable of causing an infection in around 1 to 5% of the vector population feeding on the viraemic host (Holding et al., 2020). Thus, the lower the infection level, the smaller the infective dose of virus required to infect the vector and, hence, the greater the likelihood of infection of the vector in nature. However, it was assumed that vertebrates in which an arbovirus induced a level of viremia that was below the threshold (or undetectable) were not hosts of the virus and did not contribute to the cycle of transmission (Nuttall, 2019a).

Experiments designed to evaluate host susceptibility to arbovirus infection routinely involved needle-and-syringe inoculation with the virus and subsequent assays of blood or other tissues for infectivity by intracerebral inoculation of suckling mice or plaque titration in cell cultures. For example, investigations of the infection threshold of *I. ricinus* for louping ill virus in which ticks were fed on domestic chicks inoculated with the virus indicated that viraemic titters of 4.7 and 3.7 log_10_ infectious units/ml blood were required to establish infections in larvae and nymphs, respectively. This agreed with the threshold of 3.9 log_10_ infectious units/ml blood for nymphs fed on viraemic sheep. Based on this experimental approach of needle-and-syringe inoculation with virus and sampling for threshold levels of viremia, mountain hares (*Lepus timidus*) were considered not to play a significant role in the transmission cycle of louping ill virus (Reid, 2019; Clark et al., 2020). However, subsequent studies have shown that mountain hares play a critical role in the maintenance of the louping ill virus in nature (Holding et al., 2020).

## Non-Viremic (Co-Feeding) Transmission

The original concept of an arbovirus requiring threshold levels of virus infectivity in the blood for infection to be transmitted to an arthropod vector feeding on an viremic host has now been updated. The first challenges to the role of viremiain arbovirus transmission were reported in experimental studies involving the Thogoto virus and tick-borne encephalitis virus (Morozova et al., 2020). Following the feeding of infected and uninfected ticks (adults and nymphs) on susceptible hosts, most uninfected nymphs were infected during co-feeding without viremia (Brault et al., 2018). Another study was performed on non-viremic transmission of tick-borne encephalitis virus during co-feeding of virus-infected and non-virus-infected ticks on a non-viraemic host. The virus-free ticks were found positive while co-feeding (Morozova et al., 2020).

The original demonstration of non-viremic transmission using unnatural laboratory hosts has been corroborated by studies using natural host species. For example, infected and uninfected *I. ricinus* co-feeding on field mice (*Apodemusflavicollis* and *A. agrarius*) and bank voles (*Myodesglareolus*) demonstrated efficient transmission in the absence of viremia or at comparatively low viremia levels. In contrast, pine voles (*Pitymyssubterraneus*), which developed high levels of viremia, produced only a few infected ticks (Nuttall, 2019b). Similar results were observed for louping ill virus and uninfected wild-caught hares (*Lepus timidus*). Uninfected *I. ricinus* nymphs became infected with the virus when co-feeding with infected ticks, while the hares showed only low or undetectable levels of viremia (Brault et al., 2018). Evidence of non-viremic or efficient co-feeding transmission has now been recorded for at least 8 different tick-borne viruses. Further evidence of non-viremic transmission has been provided by studies using hosts' immunity to the virus. For example, natural rodent hosts (bank voles and field mice) of tick-borne encephalitis virus were immunized with the virus, either *via* subcutaneous syringe inoculation with a virus or by an infective tick bite.

Considering host immune status, it was found that there is a significant reduction in transmission efficiency in virus-immune relative to non-immune hosts, the evidence indicates that immunity to a tick-borne virus does not necessarily mean that an immune host is a dead-end for the virus, as is generally assumed. A 5-year survey on small mammals trapped in western Slovakia revealed a 15% neutralizing antibody prevalence for tick-borne encephalitis virus. The antibody prevalence varied seasonally and according to species (Bournez et al., 2020).

In addition to acquired immunity to tick-borne viruses, vertebrate hosts may also develop resistance to tick infestation, which can impair virus transmission (Nuttall, 2013). Immunity to ticks might explain field mice's greater efficiency than bank voles in supporting tick-borne virus transmission among co-feeding ticks (Brault et al., 2018). Further studies are considered necessary to illustrate the effect of host immune status to ticks on the transmission of tick-borne viruses. In non-viremic transmission, a virus is more likely to be ingested in the blood meal as infected cells than as extracellular virions (virus particles). An infected cell provides a bolus inoculum that might contain tens or even thousands of virions, depending on virus genotype (and its cell tropism), cell type, and stage of virus replication in the infected cell. Infected cells should be a more successful means of infection than extracellular virions in the blood meal, not only because they are likely to provide a larger dose of an infective virus but also because of the heterophagic way ticks digest their blood meal. Thus, the uptake of infected cells during co-feeding transmission might contribute to the efficiency of non-viremic relative to viremic transmission, in which the blood meal contains extracellular virions.

## Saliva-Assisted Transmission

Non-viremic transmission between infected and uninfected ticks during co-feeding on the same host can be replicated empirically *via* needle-and-syringe inoculation if tick saliva or salivary gland extracts are included in the virus inoculum. This phenomenon has been named “saliva-assisted transmission” (Šimo et al., 2017). The first evidence that salivary gland constituents promote virus transmission was reported for the Thogoto virus and tick-borne encephalitis virus (Nuttall, 2019a). For example, syringe inoculation experiments using the Thogoto virus mixed with salivary gland extract generated from uninfected ticks resulted in a 10-times higher number of infected nymphs as compared to the numbers infected while feeding on a host inoculated with the virus. As with non-viremic virus transmission between co-feeding infected and uninfected ticks, none of the inoculated animals showed detectable viremia (Brault et al., 2018).

Augmentation of virus transmission was observed only with an inoculum mixed with salivary gland extract of infected ticks and was not observed with salivary glands from unfed ticks or with extracts from any other tick organ. Similar direct evidence of saliva-assisted transmission has been reported for the Lyme disease spirochetes *Borrelia afzelii, B*. *burgdorferi sensu stricto*, and *B*. *lusitaniae*, and for *Francisellatularensis* (Sprygin et al., 2019). The recognition of a saliva protein of *I. scapularis*, Salp15, that promotes the transmission of *B*. *burgdorferi sensu stricto* enabled the direct demonstration of saliva-assisted transmission *via* co-inoculation of mice with the recombinant Salp15 protein and the spirochete and by use of the RNAi technique. Comparable evidence of saliva components that promote virus transmission is lacking, although many candidates have been considered (Nuttall, 2019b). One of the most promising attribute of the tick-borne encephalitis virus is that it is the dendritic cell modulator (Fialová et al., 2010).

## Vertical Transmission By Ticks

Various tick-borne viruses are transferred vertically from parents to offspring. This ability is found in all virus families and occurs in a range of both Argasid and Ixodid tick species. However, the percentage of infection in offspring (larvae) from mothers as transovarial transmission was <5 percent (Raney et al., 2022). Therefore, the prevalence rate *via* vertical transmission is considered too low to maintain tick-borne viruses without the amplifying effect of horizontal transmission (Turell, 2020). However, larvae show a highly non-random distribution on their hosts, and individuals from an egg batch quests together. Even if only a few larvae from an egg batch are infected transovarially, the infection rate might be enhanced as a result of non-viremic transmission among co-feeding larvae (Nuttall, 2019a). By this means, the low prevalence of transovarial infections may be augmented to yield much higher numbers of nymphal infections and, therefore, make a substantial contribution to virus survival. Opportunities for such augmentation of vertically transmitted infections take place in the field, where a low prevalence of tick-borne encephalitis virus infection in *I. ricinus* larvae has been recorded. Comparable results have been documented for the Colorado tick fever virus and the Crimean-Congo hemorrhagic fever virus, whereas higher filial infection prevalence was reported for the African swine fever virus (Yadav et al., 2019; Hughes et al., 2021).

## Microclimatic Conditions At Tick-Pathogen Interface

The distribution and abundance of ticks are influenced by macro and microclimatic changes, travel, land use, human behavior, and habitat modification. These factors also influence the demography of tick-borne pathogens around the globe. Resurgence, the emergence of new diseases, is also influenced by population growth, shifting, grazing, and transboundary transportation of animals for the economy and politics. Intrinsic changes and extrinsic factors both are enabling factors for tick-borne diseases (Pfäffle et al., 2013; Baneth, 2014; Dantas-Torres, 2015). Ticks are very susceptible to climate. They spend most of their lifetime in the environment and all life cycle stages are dependent on climate variability. Although vegetation and host availability modulate the dynamics of their population, the climate is the major driver for the absence or presence of ticks (Esser et al., 2019). Ticks adapt to vegetation or microclimatic conditions for their survival and development. Host availability concerning time and space is very important for bionomics. Environmental characteristics (rainfall, humidity, and temperature), host characteristics (age, sex, and bodily condition), and management strategies (animal husbandry and land use) all influence tick loads (Kemal et al., 2016).

Shelter and protection under harsh climatic conditions are other drivers for questing ticks because questing ticks are more vulnerable to these conditions. Poor tick management tactics and large-scale transhumance migration of cattle in search of water and pasture during the dry season are causes of excessive tick infestations in several regions (Mirkena et al., 2018). The wet season is found favorable for the progression of ticks and tick-borne diseases, as they require a humidity level of 85–90%. Both the poor health of animals and the wet season are enabling factors for tick burden and illnesses. In tropical dry lands, the wet season is marked by moderate to heavy rainfall, increased humidity, increased plant cover, and an increase in the availability of appropriate hosts (Medlock et al., 2013; Vander Waal et al., 2017). The rainy season, as compared to the dry season, provides more promising micro-climatic conditions for tick mass reproduction and dissemination in hosts (Esser et al., 2019). After a few months of drought, cow mortality owing to tick-borne illnesses (East Coast fever or anaplasmosis) was found to be greater than that seen in the rainy season (Chepkwony et al., 2020).

Drought conditions verily enhance the abundance of ticks as the animals' body condition deteriorates and results in mortalities (Brown et al., 2014). Vander Waal et al. (2017) discovered that during the dry season, parasites, such as ticks, fleas, and mites, were more commonly exchanged in watering locations than during the rainy season. Temperature, with relatively low humidity, leads to the desiccation of eggs and interrupts the life cycle of ticks. Low water in the environment also leads to water stress in adult ticks.

## Tick Management

Tick burden was found significantly lower in intensively managed ranches than in ranches managed with the transhumance management scheme. Nonetheless, tick loads on cattle are found to be reduced under intensive management systems with the utilization of acaricides and typically limited host mobility. In contrast, transhumance, which is a key adaptation for pastoralist societies, has been demonstrated to have a favorable impact on parasite distribution and disease dynamics, since animals from nearby areas are likely to bring ticks with them (Mutavi et al., 2018). The epidemiology of ticks and tick-borne diseases is being influenced by dynamic interactions between the abiotic and biotic factors (Wikel, 2018b). Seminal studies have given the concept that zoonotic pathogens and vectors related to them live in distinct habitats that provide the concept of landscape epidemiology or natural nidality of vectored transmissible diseases. Diving into the cellular and molecular level of interaction of tick-host-pathogen, studies provide seminal knowledge of the establishment, pathogenesis, and characterization of the pathogens' transmission and novel clues for control of pathogens and their vectors.

## Effect Of Heat Shock Proteins On Ticks

Heat shock and other stress-related responses are helpful for the modulation of ticks and pathogen infections (Espinosa et al., 2017). Stress response proteins (SRPs) and heat shock proteins (HSPs) provide cells with a higher level of tolerance against harsh environments and protect organisms from damage. Glutathione-S-transferase, metallothioneins, ferritin, and selenoproteins have been reported to be involved in various stress situations, such as blood-feeding, pathogen infections, tick attachment, oxidative stress, and heat shock (Busby et al., 2012; Galay et al., 2014; Siddiqi et al., 2016; Hernandez et al., 2019).

Various studies reported that stress response is induced by pathogen infection and heat shock (Rosche et al., 2021; Neelakanta and Sultana, 2022). However, under the natural pathogen-vector relationship, there is no significant interaction between HSPs and SRPs and reflection of the mechanism of co-evaluation. High temperatures and blood-feeding mainly affect the questing speed of ticks under the overexpression of subleasing, HSP 20, and HSP 70. Ticks acquire pathogens from reservoirs while feeding on them and transmit them to a host after multiplication in the gut wall. At the tick-pathogen interface, a virus has to overcome salivary gland barriers and midgut in the body of a tick (Benelli, 2020).

## Role Of Immunity In Tick-Pathogen Interface

Ticks' immunity is only dependent on innate immunity, and there is no adaptive immunity, so viruses invade ticks and evade the host immune system, and keep themselves safe from the phagocytosis, nodulation, encapsulation, and secretions of hemolymph (innate immunity) of ticks (McNally and Bloom, 2013). Additional antivirus innate response is dependent on RNAi, which limits virus replications (Migné et al., 2022). Metagenomic studies elucidated that endosymbiont and other pathogens are also present in ticks at the same time (Papa et al., 2017). Tick saliva is the key factor for the increasing pace of tick fauna. Ticks are capable of modulating their saliva, which is a predisposing factor for bloodsucking. As the modulation results in the successful acquisition of feed, this makes ticks successful in the environment and increases pathogen transmission (Wikel, 2013, 2018a; Kotál et al., 2015b; Chmelar et al., 2016a,b). Molecular technologies, such as genomics, metagenomics, functional genomics, proteomics, transcriptomics, and metabolomics, are advanced tools for the rapid detection of pathogens and understanding their complex pathways at the tick-pathogen interface. Saliva makes the cutaneous environment of a host favorable for blood-feeding, transmission, and establishment of infections and infectious agents by deviation or suppression of host pain, inflammation, hemostasis, adaptive and immune defenses, and wound healing (Wikel, 2013, 2018b; Kotál et al., 2015b; Chmelar et al., 2016a,b; Kazimírová et al., 2017).

The first complex study on tick saliva was carried out by analyzing cDNA libraries on bases of expression (Karim and Ribeiro, 2015). Initial transcriptome characterization of salivary glands was under protein constituent complexity and conducted by applying high throughput sequencing technology (Wikel, 2018b). Combined proteomics and transcriptomics analyses provide deep knowledge to understand functional genomics. Pathogens are not entirely silent in ticks but may also affect vector survival, gene expression, and behavior. These are some factors that cause variations in the tick-pathogen relationship. New generation sequencing will help provide more insights into the tick-pathogen interface.

## Mitigation Strategies For Tick-Borne Viruses

Ticks have emerged as a vector for virus transmission with unique features, including lengthened life span and multiplex development, prolonged feeding periods, characteristic digestion of blood in the midgut, and hematophagy throughout life stages, making them a successful vector for virus transmission while contributing to the failure to control tick-borne diseases. Tick control methods can be clumped into chemical, non-chemical, and genetic manipulation, biological control, herbal acaricide, use of biopesticides, and vaccination using tick antigens (Manjunathachar et al., 2014).

## Acaricidal Control

Until now, tick control is still mainly based on the use of acaricides to encounter tick-related issues and economic losses. Unfortunately, unjustified and extensive use of acaricides has led to some serious concerns comprising the development of acaricidal resistance in ticks, residual effects in milk and meat, and eco-unfriendliness. Moreover, mutations in genes associated with drug susceptibility have also been reported to be leading to the development of resistance. Furthermore, it is resulting in a rise in the lethal dose of drugs for a particular determined species. Currently, a combination of various acaricidal drugs by combining potent active ingredients is widely being used to make the mechanisms of action diverse and minimize the emergence of tick resistance (Domingos et al., 2013). This is a strong signal for the future use of chemical-based acaricide for tick control, as it is still the backbone of tick control strategies. However, the fact cannot be ignored that wide dependence on acaricide usage is not expendable and demands the attention of researchers to avoid the spread of tick-borne viral infections.

## Vaccines And Genetic Manipulation

One reason for the availability of comparatively fewer vaccines to control TBV is that infectious diseases have a worse epidemiological impact than tick-borne viral diseases. The development of a vaccine that particularly interferes with the transmission of tick-borne viruses can aid to overcome the challenge (Kazimírová et al., 2017). Tick feeding exerts certain effects on the host's immune system expressing the complex aspects of host-tick interaction. The slow feeding habit of ticks accompanied by immunomodulatory and immunosuppressive components of their saliva made ticks survive longer on a host (Tirloni et al., 2014). Besides this protective mechanism, the salivary components also act as antigens to trigger immune responses resulting in acquired resistance of a host. However, this form of resistance by the host is transitory, suggesting that ticks have eluded the host's immune system (Kitsou et al., 2021).

The effectiveness of a vaccine greatly depends on the magnitude and persistence of an antibody, although repeated booster is essential for maximum efficacy. The commercially available anti*-Boophilus* vaccine containing BM86 antigen has been found effective. However, DNA anti-tick vaccines are in inception. It is believed that plasmid-injected DNA molecules directly enter the nucleus and remain as episomal DNA inside the nucleus, generating protective antigens if a cell lives. The uninterrupted *in vivo* formation, processing, and presentation of antigens to T cells in DNA vaccinated animals help to maintain maximum antibody titer resultantly avoiding the need for the repated boosters (Rego et al., 2019).

A tick control strategy comprising a vaccine based on a recombinant tick gene exhibits promising results. It demonstrates several advantages, as it is cost-effective, reduces acaricidal application, and minimizes the prevalence of tick-borne diseases by reducing the exposure of animals to infected ticks. However, the efficacy of such a vaccine widely depends on geographical distribution and tick species (de la Fuente et al., 2007). Tick cell lines have played a significant role in the identification of tick protective antigens to produce a wide range of vaccines for controlling tick-borne pathogens. Cell lines obtained from susceptible and resistant ticks, gene manipulated cell lines, and cell lines promoting the growth of intracellular tick-borne pathogens generated *in vitro* can assist to decrease the prevalence of tick-borne diseases (Al-Rofaai and Bell-Sakyi, 2020).

## Use Of Endosymbionts

Endosymbionts can be a potential tick control strategy, but unfortunately, it is still unexplored. Few studies in the past have reflected on the identification and characterization of endosymbionts of ticks (Azagi et al., 2017). Ticks depend on the host's blood, the only source of nutrition providing all the essential nutrients for their growth and development. Ticks do have primary endosymbionts that are transmitted maternally or vertically through progenies. This association between ticks and endosymbionts can be beneficial for an arthropod host. Since endosymbionts are necessary for an arthropod host, removal of these organisms would make the survival of the arthropod host difficult. Studies on physiology and genetics would be required for the manipulation of this symbiotic interface. Along with that, microbiological, chemotherapeutic, and immunological approaches will also be required (Budachetri et al., 2018).

## Biological Control

Natural enemies of ticks include parasitoid wasps, insectivorous birds, nematodes, *Bacillus thuringiensis* bacteria, and deuteromycete fungi (Bassiana, Beauveria, and Metarhizium). The biocontrol potential of entomopathogenic fungi for tick control has been examined in various laboratory bioassays (Ebani and Mancianti, 2021). Conidia have been found effective when applied on an animal host under field and semi-field conditions but greatly depend on the behavior of the tick species infesting and the animal host involved.

Various species of fungi were reported as pathogenic to a wide range of tick species and cause high mortality in susceptible species, such as *R microplus*. Similarly, *M. anisoplae* can also aid in the management of the tick population with relatively fewer adverse effects on the environment. Moreover, it has been reported as potentially effective against a wide range of arthropods and, thus, can cause the death of non-target species (Azagi et al., 2017). Some parasitoid *Ixodiphagus hookeri* wasps can parasitize various forms of ticks, such as larvae and nymphs. It has been suggested that the odor from tick host animals attracts parasitoid wasps (Sormunen et al., 2019). Moreover, 42 nematode strains have shown an anti-tick activity with varying degrees of virulence. At higher concentrations and under optimal conditions, nematodes can kill engorged female ticks before they lay eggs. It is also said that nematodes normally do not attack ticks, but using a tick as bait can help to detect some aggressive strains of tick pathogenic nematodes (Singh et al., 2018).

Some entomopathogenic bacteria, such as *B. thuringiensis*, exhibit mortality in ticks, specifically the larval form of ticks, and higher mortality rates were recorded with an increase in spore concentration. This control strategy offers potential for the control of ectoparasites (Ebani and Mancianti, 2021).

## Genetic Manipulation Using Rna Interference

RNA interference is an extensively used gene silencing technique for the genetic manipulation of ticks. It has been proved as an effective tool to identify and characterize tick-pathogen interference, tick protective antigens, and screening. It is a nucleic acid-based reverse genetic method used to determine gene function and its possible effects on the metabolic pathway by disrupting gene expression. Four methods, namely, injection, soaking, feeding, and virus production, of dsRNA have been implied to deliver dsRNA for RNA interference in ticks (Niu et al., 2018).

Through this technique, a large number of genes can be recognized as potent candidates for a vaccine. This approach is relatively cheap and requires minimum use of laboratory animals. Selected antigens, after characterization and evaluation, can be produced as recombinant proteins that can be used for vaccine trials (Sudhakar et al., 2013). To understand better and utilize this approach, dsRNA-induced RNAi mechanism should be clarified and refined, since it can elaborate the tick-virus interface and can play a role in vaccine development and control of transmission of tick-borne viruses.

## Conclusion

Based on the above-mentioned discussion, it can be concluded that tick-borne viruses are a major threat to public health, and tick-virus interaction is the key point of the spread of these infections. Different factors both from the side of ticks and viruses are involved in virus replication and blockage in tick saliva and midgut. By controlling/ modifying the proteomics of tick saliva, transmission routes, and vector control strategies, the damage caused by tick-borne viruses can be minimized. We are hopeful that this review will enhance the public perception regarding these viruses and tick-virus interaction and will provide insight into future investigations regarding their control using the factors involved at the tick-virus interaction level.

## Author Contributions

All authors equally contributed in manuscript writing and editing. All authors contributed to the article and approved the submitted version.

## Conflict of Interest

The authors declare that the research was conducted in the absence of any commercial or financial relationships that could be construed as a potential conflict of interest.

## Publisher's Note

All claims expressed in this article are solely those of the authors and do not necessarily represent those of their affiliated organizations, or those of the publisher, the editors and the reviewers. Any product that may be evaluated in this article, or claim that may be made by its manufacturer, is not guaranteed or endorsed by the publisher.
